# Supplementary material for: Learning curve and surgical outcome of robotic assisted colorectal surgery with ERAS program
Source: Sci Rep. 2022 Nov 29;12:20566. doi: 10.1038/s41598-022-24665-w (PMC9709162; doi:10.1038/s41598-022-24665-w)
Supplement: Supplementary file 1 — Supplementary Information 1. [file 41598_2022_24665_MOESM1_ESM.zip › LSC Raw data-Ñ[▒K/064/O_ñw╝╨░O▒Kñσ.pdf]

正式摘要(電子簽章：000000185462308)

W86 / W86

臺中榮民總醫院

Operation Note

[redacted]

- 手術日期：2020/02/06
- 手術醫師：VS 蔣鋒帆，CR 許家榮，CR 黃偲霖，R 楊涵中，R 陳思如
- Anesthesia : GE
- Position : Lithotomy
- Aseptic agents : Betadine Alcohol , Betadine Aqua

Diagnosis

- Pre-operative diagnosis : Malignant tumor of rectum, 7cm FAV with liver metastasis
- Post-operative diagnosis : Ditto. s/p laparoscopic low anterior resection with end to end anastomosis

Surgical Indication

- for resection and pathologic staging

Operation

- 1.Laparoscopic low anterior resection with end-to-end anastomosis, double stapling, EndoGIA 45/4.1mm*2, CDH 29mm, bilateral doughnuts: intact, air leak test: done with air leak.
  2. High ligation (+) above the left colic artery.
  3. Cut margin: proximal: 15 cm, distal: 3 cm.
  4. Take down of splenic flexure: nil
  5. Drain: a 12mm penrose drain in pelvis
  6. Repair of mesentery defect:nil

Finding

- 1.One Borrmann's type II ulcerative tumor, measured about 2.5 x 3 cm in size, located at middle rectum, which invaded through muscularis propria. No enlarged LN noted. Clinical stage: T3N0M1a
  2.Liver tumor over S4 was noted

Procedure

- General endotracheal anesthesia was induced and then the patient was placed in modified Trendelenburg lithotomy position. Preoperative antibiotics were given. A Foley catheter and a nasogastric tube were placed. The abdomen was prepped and draped in standard sterile fashion. A small vertical incision was made beside the umbilicus and a 12mm trocar was inserted into the peritoneal cavity by open method. Peumoperitoneum was established with CO2 inflation. Laparoscopy was inserted and then another 12mm trocar was inserted over RLQ. Three 5mm trochars were inserted over RUQ, LUQ and LLQ of abdomen. The operative findings were mentioned as above. The small bowel was inspected and retracted to the right by grasper.
  The sigmoid colon was then held by grasper and the peritoneum overlying the mesentery was then scored with electrocautery. The inferior mesentery artery was identified, ligated above the left colic artery with endoclips, and transected, so was inferior mesentery vein. Dissection was done above the Gerota's fascia and toward the peritoneal attachments. The plane anterior to the aorta was entered and colon, mesenteric fat were mobilized from retroperitoneum overlying the sacral promontory and into the pelvis. The incision was extended laterally and medially across the peritoneal reflection. The mesorectum was then mobilized using sharp and blunt dissection. Care was taken not to enter the presacral venous plexus. The lesion was palpable and the rectum was carefully elevated until an adequate distal margin was noted. A linear stapler was then used to divide the lower rectum.

- One small midline incision was made. The proximal colon was carefully inspected, noted to reach easily into the pelvis, and to have an excellent blood supply. The mesenteric fat was gently cleared from the terminal 2-3 mm of the bowel ends. The field was isolated with moist pad and the bowel opened and sized. It was determined that a 29 mm circular stapler could be accommodated. A pursestring suture of 2-0 Nylon was placed, the anvil was inserted, and then the pursestring suture was tied. Pneumoperitoneum was acheived again. The anal canal was irrigated with distil water and then the circular stapler was introduced through the anus and pierced through the mid-portion of the stapler line. The proximal bowel with anvil in place was engaged with the circular stapler and the stapler was then fired. Two complete donuts were obtained and submitted to pathology. The anastomosis was tested for leaks with irrigation solution and air introduced through the rectum and was found leakage. A 12mm penrose drain was placed in pelvis. The fascia of midline wound was closed with interrupted sutures of No.1 Vicryl. The fascia of the 12 mm trochar was closed with 1-0 Vicryl. The skin wounds were closed with 3-O Nylon. The patient tolerated the procedure well and was brought to the POR in stable condition.
- Estimated blood loss: minimum
- 器械數量: 正確
- 紗布數量: 正確

Image

- PAINT
  - colon & rectum

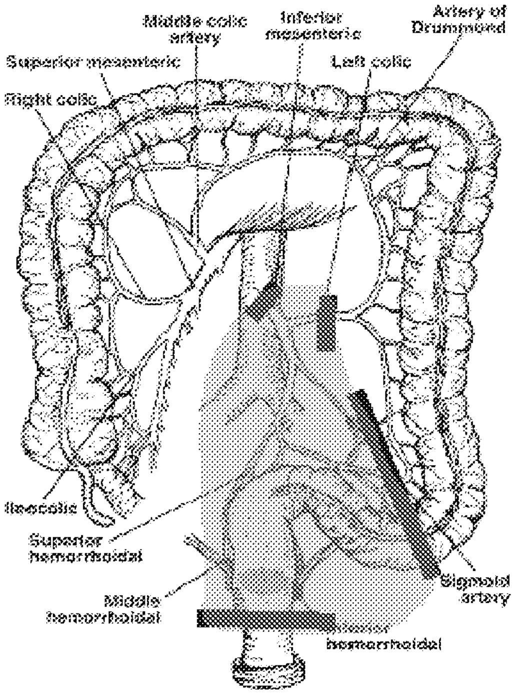

  - abdomen

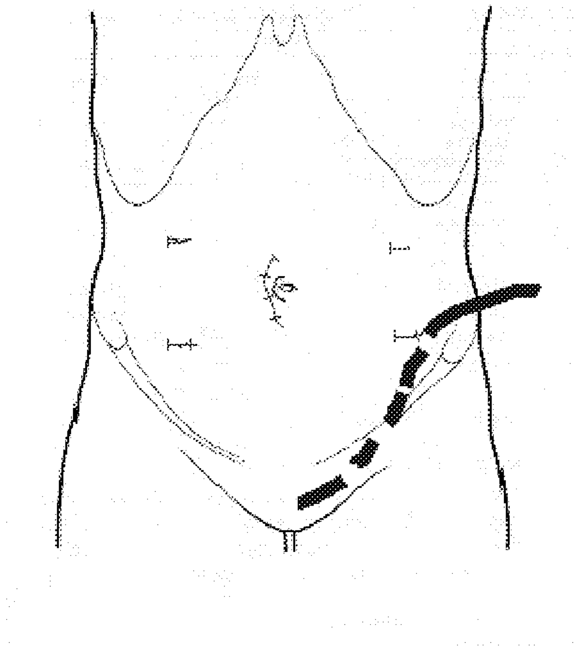

主任 2062F蔣鋒帆 / 醫師 2319H許家榮

正式摘要(電子簽章：000000185462308)

	W86 / W86

doctorRank:B
